# Neural Circuitry of Impulsivity in a Cigarette Craving Paradigm

**DOI:** 10.3389/fpsyt.2013.00067

**Published:** 2013-07-16

**Authors:** Josiane Bourque, Adrianna Mendrek, Laurence Dinh-Williams, Stéphane Potvin

**Affiliations:** ^1^Centre de Recherche de l’Institut Universitaire de Santé Mentale de Montréal, Montréal, QC, Canada; ^2^Department of Psychiatry, Faculty of Medicine, University of Montreal, Montreal, QC, Canada; ^3^Department of Psychology, Bishop’s University, Sherbrooke, QC, Canada

**Keywords:** craving, impulsivity, cigarette smoking, neural correlates, fMRI

## Abstract

Impulsivity has been shown to play a pivotal role in the onset, pattern of consumption, relapse and, most notably, craving of illicit and licit drugs such as cigarette smoking. The goal of this study was to examine the neurobiological influence of trait impulsivity during cue-induced cigarette craving. Thirty-one chronic smokers passively viewed appetitive smoking-related and neutral images while being scanned and reported their feelings of craving. They completed the Barratt Impulsiveness Scale, a measure of trait impulsivity. We conducted functional connectivity analyses using the psycho-physiological interaction method. During the processing of smoking stimuli, participants presented increased activations in the cingulate and prefrontal cortices. We observed a significant positive relationship between impulsivity scores and reported craving. A negative correlation was observed between the impulsivity score and activity in the posterior cingulate cortex (PCC). The insula, dorsal anterior cingulate cortex (dACC) as well as the dorsolateral prefrontal cortex (DLPFC) presented a negative connectivity with the PCC. Consistent with the view that the PCC is related to the ability to resist cigarette craving, our results suggest that high impulsive smokers have greater difficulty in controlling their cravings, and that this weakness may be mediated by lower PCC activity. Moreover, we argue that the less PCC activity, the greater the probability of a stronger emotional, physiological, and biased attentional response to smoking cues mediated by insula, dACC, and DLPFC activity. This is the first study on this topic, and so, results will need to be replicated in both licit and illicit drug abusers. Our findings also highlight a need for more emphasis on the PCC in drug addiction research, as it is one of the most consistently activated regions in functional magnetic resonance imaging studies examining the neural correlates of cue-induced alcohol, drug, and tobacco cravings.

## Introduction

Approximately 20% of adults in Canada and in the US smoke tobacco, and are at high risks (a three to ninefold increased risk relative to the general population) of developing cancer or pulmonary and cardiac diseases (1–3). Given its deleterious effects on health, more than half of cigarette smokers express a desire to quit (4). Unfortunately, 72–90% of smokers attempting to quit, with or without treatment, will have relapsed by 1-year following their quit date (5–7). Cessation attempts and treatment methods would be greatly ameliorated by improving understanding of the behavioral and neurobiological mechanisms underlying relapse.

Clinical research consistently demonstrates that one of the best predictors of smoking and illicit drug abuse relapse is craving (8, 9). Craving is a multifaceted state characterized by both automatic and non-automatic processing, the latter involving cognitive efforts to either aid or prevent the execution of automatized sequences of drug use (10–12). In abstinent and non-abstinent smokers, exposure to cigarette-associated cues and/or stress can induce a strong craving response, and therefore enhance the risk of relapse in abstinent smokers (8, 13, 14). Over the past decade, there has been a growing interest in examining the biological roots of cue-elicited cigarette craving using functional magnetic resonance imaging (fMRI). Recent review and meta-analysis have shown that overall, in both deprived and non-deprived smokers, cigarette craving is associated with cerebral activations in the extended visual system, the superior and middle temporal gyri, the precuneus, the posterior and anterior cingulate gyri, the prefrontal and orbitofrontal cortices, the insula, as well as the dorsal striatum (15, 16). However, when looking at each individual study, we found that several factors can contribute to the heterogeneity of findings *between* studies, notably smoking expectancy and abstinence levels (17–21).

Moreover, there is also a strong between subject variability *within* studies in the overall brain activations associated with exposure to smoking cues (22) and more specifically, in terms of the peak activity location for each participants (23). It is crucial to further understand this variability in brain reactivity to cigarette stimuli and craving response. To our knowledge, only nicotine dependence severity, sex, one’s degree of discontent toward his/her smoking behavior and genetic factors (e.g., dopamine transporter) have been studied as potential factors implicated in cigarette craving brain responses (22, 24–26). For instance, nicotine dependence severity was associated with specific cerebral activations in four out of five studies (24, 25, 27–29). In the present study, we wished to examine if the reward-related personality trait of impulsivity, which has been shown to play a key role in addiction (cigarette, alcohol, cocaine), influences the pattern of brain activity observed during an appetitive smoking-related state.

Impulsivity traits have been (1) consistently associated with drug abuse and smoking behavior. Current smokers compared to non- and former-smokers present higher scores of impulsivity (30, 31). In addition, cognitive measures of impulsivity such as inattention, disinhibition, and impulsive decision-making are related to smoking status in both adolescents and adults (30, 32, 33). Impulsivity has also been shown to be (2) a good predictor of the onset and increase of substance use in early adolescence (34, 35). In the case of smoking, results have been less consistent, however a number of studies have suggested that hyperactivity/impulsivity predict tobacco smoking in adolescents (36). Finally, (3) smoking relapse is influenced by personality traits. Several studies have reported that following a 1- to 2-month cessation program, highly impulsive adolescent smokers were less successful in quitting than non-impulsive smokers (37, 38). Furthermore, experimental studies have shown that during abstinence, those with high levels of trait impulsivity present significantly greater cravings and positive reinforcement expectancies from smoking, relative to low impulsive smokers (39–41).

Therefore, the main purpose of this study was to explore the association between trait impulsivity and the neural correlates of cue-elicited cigarette craving. To our knowledge, this is the first study to investigate this topic. We examined impulsivity as a broad construct, such as a personality trait, rather than components that reflect impulsivity (e.g., response inhibition). Among the brain regions involved in nicotine craving, the dorsolateral prefrontal cortex (DLPFC), the orbitofrontal cortex (OFC) as well as the dorsal anterior cingulate cortex (dACC) all play a significant role in self-control processes, such as executive control and adaptive responding. These structures have been shown to be functionally impaired in substance abusers and cigarette smokers (42–46) and thus, have been hypothesized as important structures in the maintenance of addictive behavior. Additionally, various regions of the cingulate cortex have been directly implicated in resisting cigarette craving (47–49). Despite the exploratory nature of this work, we expected to find a positive correlation between cue ratings of craving and impulsivity; a negative correlation between trait impulsivity and brain regions underlying impulse control; and a negative coupling (functional connectivity) between impulse-control areas and brain regions responsible for attributing salience to smoking-related cues.

## Materials and Methods

### Participants

Thirty-one healthy smokers (15 men) were recruited through the research center and affiliated hospital, as well as using Internet advertisements. Participants were chronic smokers (between 12 and 33 cigarettes/day; mean: 19.3; SD: 5.7) not currently seeking treatment, aged 18–55 years old (mean: 31.8; SD: 9.2), right-handed (except for 1 ambidextrous and 1 left-handed), Caucasian (84%), with no concomitant neurological, axis I or axis II disorder (based on self-report of previous diagnosis); and no contra-indications for MRI. The average number of years of education was 12.9 (SD: 2.7). None of the participants received psychiatric or neurologic drug treatment. Participants had been smoking cigarette for an average of 15.9 years (SD: 9.6) prior, with their first cigarette at 16.0 (SD: 3.4) years of age, and had tried quitting an average of 2.7 times (SD: 2.7).

Nicotine dependence severity was assessed using the Fagerström Test for Nicotine Dependence (FTND) (50), and cigarette craving with the French Tobacco Craving questionnaire (FTCQ-12) (51) prior to scanning. In addition, our group was required to fill the Readiness to Quit Ladder questionnaire (52) on a scale from 1 (I have decided not to quit smoking for my lifetime. I have no interest in quitting) to 10 (I have quit smoking); participants were instructed to choose the number that represents his or her present state best. We administered the Beck Depression Inventory (BDI) (53) and the State-Trait Anxiety Inventory (STAI) (54) as measures of depression and anxiety symptoms respectively. Lastly, the participants completed the Barratt Impulsiveness Scale (BIS-11), a measure of impulsive personality traits (55).

In agreement with the *Declaration of Helsinki*, written informed consent was obtained from each participant prior to the testing sessions. The study was approved by the ethics committee of the *Réseau de Neuroimagerie du Québec*.

### fMRI procedure

Thirty to 40 min prior to each fMRI scanning session, participants were invited to smoke a cigarette to minimize withdrawal effects and standardize the period of non-smoking. While in the scanner, following the anatomical acquisition, participants passively viewed an alternating sequence of appetitive smoking-related images from the International Smoking Image Series (ISIS) (56) and neutral pictures taken from the International Affective Picture System (IAPS) (57). Neutral IAPS pictures were matched with the smoking-related images (ISIS) for visual complexity, color, and number of faces and body parts.

During the scanning session, participants were instructed to press a button when a picture appeared in order to monitor their level of attention. The task consisted of an alternating sequence of five experimental (appetitive smoking-related images) and five control condition blocks (neutral pictures) with 10 periods of rest separating the blocks from one another. The rest period consisted of a 15-s blank screen with a fixation cross. Each block lasted 25 s and consisted of five pictures, presented for 4 s each. There was an inter-stimulus interval (blank screen) of an average of 1 s (ranging from 0.5 to 1.5) presented before each picture. Within a block, images were randomly presented. Participants viewed a total of 25 appetitive as well as 25 neutral pictures.

At the end of the fMRI session, participants were re-presented with the smoking-related and neutral images, and were asked to rate them on a scale from 0 (images elicit no desire to smoke a cigarette) to 100 (images elicit the strongest desire to smoke ever experienced).

### fMRI data acquisition

We recorded blood oxygenation level dependent signals using a single-shot, gradient-recalled echo-planar imaging sequence (repetition time = 3000 ms, echo time = 30 ms, flip angle = 90°, matrix size = 64 × 64 voxels, field of view = 224 mm, number of slices = 41, slice thickness = 3.5 mm, interslice gap = no gap, voxels size = 3.5 mm × 3.5 mm × 3.5 mm) on a Siemens TRIO MRI system at 3.0 Tesla at the *Functional Neuroimaging Unit at the University of Montreal Geriatric Institute*. We then registered the whole brain functional volumes to individual high-resolution co-planar anatomical images taken during the same scanning session (three-dimensional, ultrafast gradient echo sequence; repetition time = 2300 ms, echo time = 2.98 ms, flip angle = 9, matrix size = 256 ×  256 voxels, number of slices = 176, voxels size = 1.0 mm × 1.0 mm × 1.0 mm).

### fMRI data analysis

We analyzed fMRI data using a statistical parametric mapping software (SPM5: Wellcome Department of Cognitive Neurology, London, UK) according to the methods outlined by Friston (58). The functional images were realigned to the mean volume of the run to correct for artifacts due to minor head movements, high-pass filtered, spatially normalized into the standardized T1 brain template, and spatially smoothed with a three-dimensional isotropic Gaussian kernel (8 mm FWHM) to improve signal-to-noise ratio.

We used a standard peak-detection approach and the general linear model implemented in SPM5 for our statistical analyses in order to identify the dynamic cerebral changes associated with cigarette craving, using a block design. First, we undertook a fixed-effects analysis for each participant to investigate individual brain activation maps associated with our contrasts of interest (appetitive smoking-related minus neutral material, and vice versa). A second-level random-effects model was then implemented to investigate the pattern of activations during both contrasts (i.e., appetitive minus neutral material and neutral minus appetitive material) in our group, using a one-sample *t*-test. We performed region-of-interest (ROI) analyses, using the “small volume correction” option of SPM at a threshold of *p* < 0.05, false discovery rate (FDR) corrected for multiple comparisons. The small volume was chosen using a sphere (radius = 12 mm) located in the center of the corresponding region according to the *Automated Anatomical Labeling* atlas (59). Our ROIs included the medial and DLPFC; anterior, middle, and posterior cingulate gyri; OFC; dorsal striatum; precuneus as well as the insula. In addition, using the Volume of Interest tool in SPM, we extracted the first eigenvariate from the one-sample *t*-test (for the contrast: appetitive smoking-related minus neutral material) based on the center of each ROI clusters identified by SPM. We then performed Pearson correlation analyses with the *Statistical Package for the Social Sciences* (SPSS) to investigate the association between the impulsivity score from the BIS-11 and first eigenvariates of each ROI, and also between cue ratings of craving and first eigenvariates of each ROI. To investigate the extensive relationship between impulsivity and ROI activations, we used the psycho-physiological interaction (PPI) method, a multiple regression technique that allows the investigation of the functional coupling between regions in relation to the experimental paradigm (60). Consequently, for each ROI (those significantly correlated with the impulsivity score) we extracted the first eigenvariate time series from each participant using the Volume of Interest tool in SPM. The PPI regressor was calculated as the element by element product of the ROI time series and a vector coding for the effect of task (craving minus neutral condition). This interaction term was then entered as a regressor of interest in a first level model together with the ROI time series and the vector coding for the task effect. Ultimately, model contrasts were generated to test the effects of positive and negative PPIs. Given the paucity of studies investigating functional connectivity in addiction, we set the threshold level for statistical significance at *p* < 0.001 (uncorrected). For all types of analyses, we considered extent thresholds of 20 contiguous voxels.

### Behavioral data analyses

We performed Pearson correlation analyses between the impulsivity score (total and second order factors) on the BIS-11 and ratings of craving with SPSS.

## Results

### Self-report

As displayed in Table 1, participants presented minimal symptoms of anxiety and depression. In general, participants were moderately dependent on nicotine, had moderate level of cravings and their motivation to quit corresponded to: I often think about quitting smoking, but I have no plans to quit. Finally, they presented a mean impulsivity level in normal limits. When looking at the appetitive smoking images from the task, participants rated the intensity of their craving at 47.1% (SD: 27.9) of strongest desire ever experienced.

**Table 1:** **Demographic data. Standard deviation (SD) in parentheses**.

Questionnaires	Mean results
STAI (State-trait anxiety inventory)	35.6 (SD: 8.0)
BDI (Beck depression inventory)	5.3 (SD: 6.0)
FTND (Fagerström test for nicotine dependence)	4.3 (SD: 2.6)
FTCQ-12 (French tobacco craving questionnaire)	3.6 (SD: 1.0)
Readiness to quit ladder	5.2 (SD: 1.3)
BIS-11 (Barratt impulsiveness scale)	63.7 (SD: 10.8)

We observed a significant positive correlation between the total impulsivity score on the BIS-11 and the reported craving from the smoking-related images (*r* = 0.624; *p* < 0.001). Among second order factors (attentional, motor, and non-planning) of the BIS, it was the non-planning subscale which was the most significantly positively correlated with the ratings of craving (*r* = 0.625; *p* < 0.001).

### fMRI

#### One-sample *t*-test

**Figure 1:**
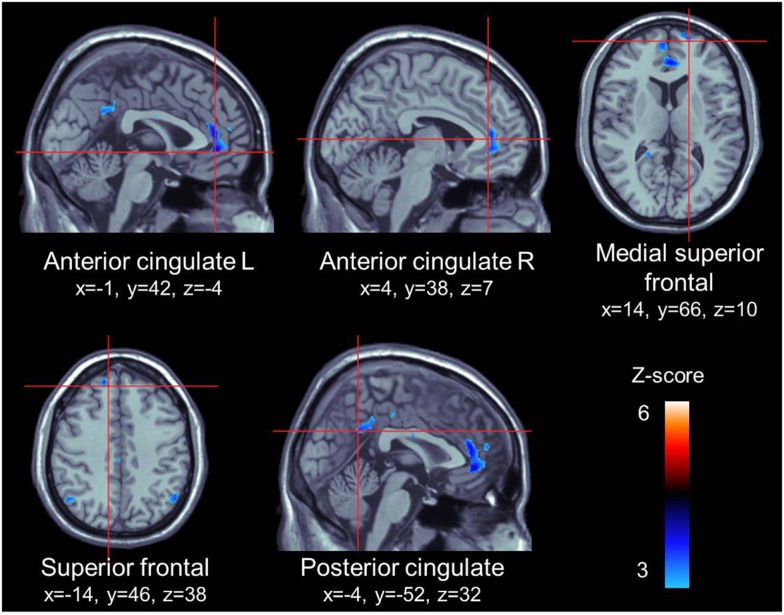
**ROI activations during viewing of appetitive cigarette images (relative to neutral images)**. All highlighted regions in this figure were significantly activated. Transition from blue to red color represents increases in*z*-scores.

For the appetitive smoking-related minus neutral contrast, ROI analyses revealed significant loci of cerebral activations in the anterior cingulate gyrus bilaterally, the right medial superior frontal gyrus, the left superior frontal gyrus, and the left posterior cingulate gyrus. Conversely, we found no significant loci of activations in our ROIs for the neutral minus appetitive smoking-related contrast (Table 2; Figure 1).

**Table 2:** **ROI activations during viewing of appetitive cigarette and neutral images**.

Brain region	R/L	BA	MNI coordinates			*z*-Score	Voxels	*p*-Value
			*x*	*y*	*z*			
**SMOKING > NEUTRAL**								
Anterior cingulate gyrus	L	32	−1	42	−4	3.54	170	0.008
	R	24	4	38	7	3.48	170*	0.008
Medial superior frontal gyrus	R	10	14	66	10	3.43	24	0.025
Superior frontal gyrus	L	9	−14	46	38	3.32	24	0.038
Posterior cingulate gyrus	L	31	−4	−52	32	3.27	84	0.021

R, right; L, left; BA, Brodmann area; *same cluster as above; p-value is FDR corrected at<0.05.

*Neutral > smoking*.

*No significant activations in the ROIs*.

#### Correlations

**Figure 2:**
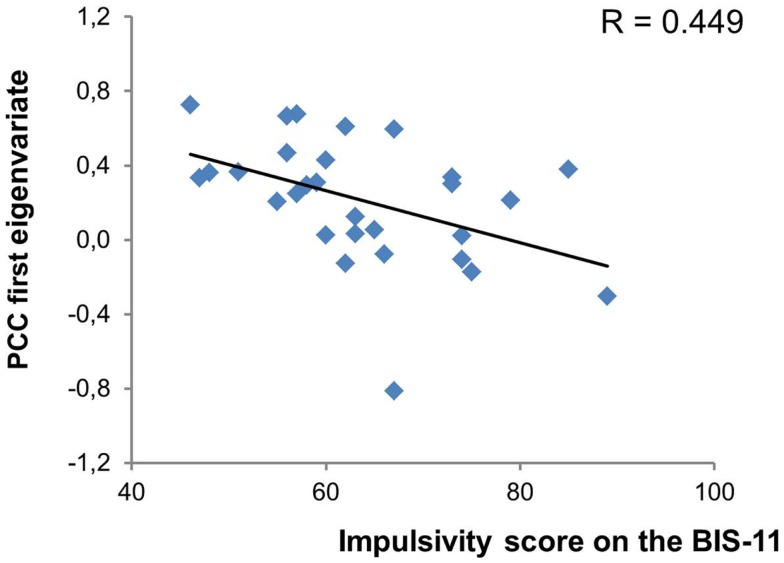
**Significant relationship between impulsivity levels and posterior cingulate cortex activity**. PCC, posterior cingulate cortex.

We found no correlation between cue ratings of cravings and activations in any of our ROIs for the appetitive smoking minus neutral contrast. However, as shown in Figure 2, there was a significant negative correlation between the total impulsivity score on the BIS-11 and activity in the left posterior cingulate gyrus (*r* = −0.449; *p* = 0.015). More specifically, among the second order factors of impulsivity only the non-planning subscale presented a significant negative relationship with the posterior cingulate gyrus (*r* = −0.440; *p* = 0.017). The posterior cingulate gyrus was therefore used as the seed region for the PPI analyses. Impulsivity (total and subscale scores) was not correlated with any other brain region.

#### Psycho-physiological interaction

**Figure 3:**
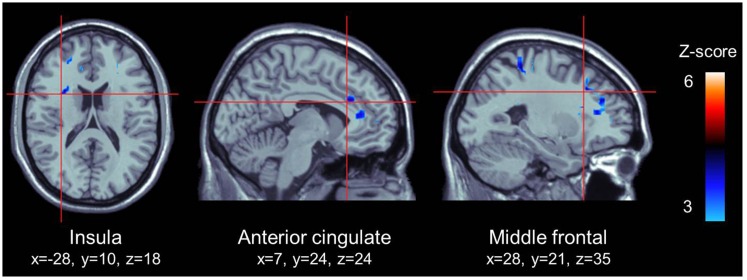
**ROI that presented a significant negative connectivity with the posterior cingulate cortex**. All highlighted regions in this figure were significantly activated. Transition from blue to red color represents increases in *z*-scores.

We used the PPI method to explore the functional coupling of the posterior cingulate gyrus, and found that the left insula (MNI coordinates: *x* = −28; *y* = 10; *z* = 18; 126 voxels; *z* = 3.81; *p* < 0.001), the right middle frontal gyrus [MNI coordinates: *x* = 28; *y* = 21; *z* = 35; Broadmann area (BA) = 9; 227 voxels; *z* = 3.74; *p* < 0.001] and the right dACC (MNI coordinates: *x* = 7; *y* = 24; *z* = 24; BA = 24; 25 voxels; *z* = 3.60; *p* < 0.001) presented significant negative connectivity with the posterior cingulate gyrus, as shown in Figure 3. There were no significant positive connectivity between the posterior cingulate gyrus and any region of the brain.

## Discussion

As craving and impulsivity traits are both strong predictors of relapse, the aim of the present study was to explore the neural correlates underlying their relationship in chronic tobacco smokers. Similarly to previous neuroimaging studies on drug cue-reactivity, we reported significant activations in regions of the cingulate and prefrontal cortices during the processing of appetitive smoking-related stimuli (15, 16). We also found important correlations between impulsivity traits and both behavioral and neurobiological measures of craving. Accordingly, impulsivity was (1) positively correlated with the reported craving from the fMRI task and (2) negatively associated with the activity of the posterior cingulate cortex (PCC) during cigarette cue response. Finally, functional connectivity analyses revealed that the activity of the insula, DLPFC and dACC were negatively correlated with that of the PCC during cue-elicited craving.

The processing of appetitive cigarette smoking relative to neutral stimuli elicited significant loci of activations distributed in regions of the cingulate gyrus (bilateral ventral part of the anterior cingulate cortex and left PCC) and the prefrontal cortex (right medial superior and left superior frontal cortices). The anterior cingulate and prefrontal cortices are the two most consistently reported structures in cigarette craving studies (16). The ventral part of the anterior cingulate cortex, known as the affective region, is involved in regulating emotional responses (61) and more specifically, assessing the salience of emotional and motivational information, such as drug-related cues (62). The medial prefrontal cortex is widely implicated in self-related processes (63, 64) and of note, various authors have found that self-referential processing of cues (medial prefrontal cortex activity) enhances learning and promotes behaviors oriented toward the content of these cues (65–67). Therefore, it is possible that the activity observed in the medial prefrontal cortex during smoking cues reflects self-related processing, and promotes smoking behavior. The PCC has been repeatedly activated during cue-elicited cigarette craving, but have received far less attention (16). This region has been linked to attentional tracking of stimuli, memory recall and, similarly to the medial prefrontal cortex, self-referential, and reflective activity (16, 63, 68, 69). Thus, the response to smoking cues observed in this study may be reflective of smokers’ attentional bias toward smoking cues, its self-relevance and emotional salience, memory of its incentive value, and preparing for the physical act of smoking.

On a behavioral level, the total score of impulsivity presented a strong positive relationship with the cue ratings of craving. These results are in accordance with a vast array of clinical and experimental studies showing a positive correlation between impulsivity and alcohol (70–72), nicotine (73, 74), as well as cocaine and methamphetamines craving (75). What’s more, between the three different second order factors of the BIS-11, the non-planning factor had the strongest association with the reported craving. As this factor involves a lack of reflection, self-control, and forethought (76), our results corroborate the importance of these cognitive factors in addictive behavior and a craving response. Indeed, relative to conditioning-based models of craving, cognitive views emphasize the necessity of non-automatic processes for overriding automatic drug use patterns (e.g., to halt the execution of automatized drug use acts) (10, 11). Therefore, we suggest that increasing levels of trait impulsivity and poor planning abilities/self-control in cigarette smokers is related to diminished capacities to suppress these automatic drug thoughts and urges.

Our neuroimaging results demonstrated no direct association between subjective craving and brain activations during the processing of cigarette cues (versus neutral images). Nevertheless, we observed a strong negative relationship between the total score of trait impulsivity and activity in the left PCC during response to smoking stimuli (versus neutral images). Among the second order factors of the BIS-11, non-planning covaried negatively with the PCC. This result suggests that increasingly poor levels of self-control and self-reflection are associated with decreased fMRI signal in the PCC, compared to less impulsive smokers. Consistent with our finding, the PCC is related to the ability to resist cigarette cravings. Brody et al. (47) found that activity in the left PCC was greater while participants were actively trying to suppress their urge to smoke than while allowing themselves to crave. It is possible then that individuals with poor planning abilities/self-control experience greater difficulty in controlling their cravings, and that this weakness may be mediated by lower PCC activity. Of note, we did not observe any direct relationship between impulsivity measures and activity in the DLPFC or OFC, which are commonly reported as centers of impulse control in studies examining the neurophysiologic basis of impulsivity, using decision-making or response inhibition tasks (77, 78). These studies however also report activity in the PCC during these tasks (79–81). Research has shown, for instance, that increased activity in the PCC during behavioral measures of impulsivity, such as a Go/NoGo task, is related to response inhibition (80, 81), but further investigation revealed that the PCC function was specific during incorrect NoGo versus correct ones (79). Moreover, it must be considered that fMRI studies on decision-making and response inhibition measure small-scale behavioral components of impulsivity rather than habitual patterns of behavior, thought, and emotion. It is possible that impulsivity as a trait refers to more complex neural processes (e.g., interaction between the PCC and DLPFC) than those implicated in transient decision-making or response inhibition events. Importantly, the PCC has been systematically reported in fMRI meta-analytic studies of cigarette, alcohol, and illicit drug craving (16, 82–84). Highly impulsive individuals are stimulus-bound and focus on the immediate environment and circumstances rather than long term events. This behavior illustrates a lack of introspection and mentalizing. The PCC is critical, over and above the OFC and DLPFC, for self-relevant processes such as mindfulness, mentalization, and self-reflection that promote mental exploration as well as build cognitive and affective control (85–87). Furthermore, an altered functional connectivity of the PCC with other frontal regions was demonstrated in psychiatric disorders characterized by high levels of impulsivity (88–90). The PCC may be a key structure underlying impulsivity and warrants more attention.

To further explore the mechanisms underlying the relationship between impulsivity/non-planning and the PCC, we conducted functional connectivity analyses. Using a liberal statistical threshold, the PPI analyses revealed significant negative connectivity between the PCC and the left anterior insula, the right DLPFC (BA 9) as well as the right dACC (BA 24). Noteworthy, the left anterior insula was activated during the appetitive smoking-related versus neutral contrast, but not reported here in the present study due to a low cluster threshold (> 5 contiguous voxels). The more anterior parts of the insula are specifically recruited during the regulation of the physiological and emotional body response to stimuli (91, 92), while the DLPFC and dACC are directly implicated in allocating and coordinating attentional resources for cognitive tasks (93–95). We argue that the more impulsive the smoker, the greater the probability of a decrease in PCC activation and increase in insula, DLPFC, and dACC activity. This may result in a decrease of the self-reflection processes necessary for self-control and may intensify the emotional, physiological, and biased attentional response to smoking cues. Indeed, the PCC and the dACC are anatomically linked by the cingulum bundle, an important white matter fiber tract connecting limbic-cortical networks. Although the literature on functional connectivity in addiction is limited, a few preliminary studies have shown a negative relationship between the activity of the PCC and dACC. Both methamphetamine abusers and chronic smokers present inversed activations/glucose metabolism in the PCC and dACC while executing vigilance tasks (96, 97). Similarly, some authors have underlined an abnormal connectivity between the PCC and the anterior part of the insula in heroin addicts (98) as well as between the PCC and DLPFC in recently abstinent cocaine addicts during negative emotional experience (100). Altogether, these preliminary results propose a neural pathway for an exacerbated feeling of craving in highly impulsive cigarette smokers.

Overall, the results of our study may have implications for the understanding of the neural relationship between the reward-related personality trait of impulsivity and cue-elicited craving. Importantly, both impulsivity and craving are phenotypes that have become major targets of psychological, pharmacological, and brain stimulation interventions in the field of addiction (46, 99, 101, 102), and the brain stimulation literature has recently raised the possibility that the mechanisms underlying impulsivity and craving may partially overlap (46, 103). However, a few limitations must be considered. Indeed, the PPI results need to be cautiously interpreted because these analyses do not allow determining the direction of the relationship between the PCC and the insula, DLPFC as well as the dACC. Here, the activity of the PCC was negatively correlated with the activity of these structures, however we do not know if it is the reduced activity within the PCC observed in highly impulsive smokers that lifts inhibition toward these structures, or it is the activation of the insula, DLPFC, and dACC that down regulate PCC function. Additionally, both the dACC and DLPFC, which presented a negative connectivity with the PCC were not significantly activated during smoking cue responses. One plausible explanation is that our participants had not been abstinent overnight for the study (they smoked 30–40 min prior to the fMRI session). For instance, important evidences have underlined that compared to relatively non-deprived smokers, overnight deprived smokers present significantly greater activations, amongst other regions, in the DLPFC and regions of the cingulate cortex (16, 21). Finally, the brain response to smoking cues could, in part, be influenced by the participants’ simple motor task (i.e., press of a button) during viewing of appetitive and neutral pictures (104). However, here, we did not observe any activation in premotor and motor areas, even in exploratory analyses.

Although there is a vast array of clinical and experimental literature on the relationship between impulsivity traits and drug craving, this is the first study to examine the neural correlates underlying their relationship. This exploratory work reveals the neurobiological mechanisms by which impulsive traits influence the brain response to smoking cues. As impulsivity is both a solid indicator of relapse and a core feature of smoking behavior, our findings are relevant from a cessation treatment point of view. In effect, present results combined with several other findings support the necessity of treatment interventions based on reward-related personality traits such as impulsivity to alleviate feelings of craving and propose a potential pathway, that needs to be investigated further, by which impulsivity and craving interact. For the moment, our results will need to be replicated in chronic smokers as well as in patients abusing other psychoactive substances, and should encourage further research on the PCC in drug addiction, which remains poorly understood at the moment, although it is one of the most consistently activated regions in fMRI studies examining the neural correlates of cue-elicited alcohol, drug, and tobacco cravings.

## Conflict of Interest Statement

The authors declare that the research was conducted in the absence of any commercial or financial relationships that could be construed as a potential conflict of interest.
